# Arsenic in Drinking Water and Mortality for Cancer and Chronic Diseases in Central Italy, 1990-2010

**DOI:** 10.1371/journal.pone.0138182

**Published:** 2015-09-18

**Authors:** Daniela D’Ippoliti, Enrica Santelli, Manuela De Sario, Matteo Scortichini, Marina Davoli, Paola Michelozzi

**Affiliations:** Department of Epidemiology, Lazio Regional Health Service, Rome, Italy; University of Louisville, UNITED STATES

## Abstract

**Background:**

In several volcanic areas of Italy, arsenic levels exceed European regulatory limits (10 μg/L in drinking water). There is still uncertainty about health risks from arsenic at low-medium doses (<100 μg/L).

**Objectives:**

A large population-based study using an administrative cohort of residents in the Viterbo province (Central Italy), chronically exposed to low-medium arsenic levels via drinking water, was investigated to evaluate the effects of a lifetime exposure to arsenic on mortality from cancers and chronic diseases.

**Methods:**

The study population consisted of 165,609 residents of 17 municipalities, followed from 1990 until 2010. Average individual arsenic exposure at the first residence (As_I_) was estimated through a space-time modeling approach using residential history and arsenic concentrations from water supply. A time-dependent Cumulative Arsenic dose Indicator (CAI) was calculated, accounting for daily water intake and exposure duration. Mortality Hazard Ratios (HR) were estimated by gender for different diseases using Cox proportional models, adjusting for individual and area-level confounders. A flexible non-parametric approach was used to investigate dose-response relationships.

**Results:**

Mean As_I_ exposure was 19.3 μg/L, and average exposure duration was 39.5 years. Associations of As_I_ and CAI indicators with several diseases were found, with greatest risks found for lung cancer in both sexes (HR = 2.61 males; HR = 2.09 females), myocardial infarction, peripheral arterial disease and COPD in males (HR = 2.94; HR = 2.44; HR = 2.54 respectively) and diabetes in females (HR = 2.56). For lung cancer and cardiovascular diseases dose-response relationship is modelled by piecewise linear functions revealing effects even for doses lower than 10 μg/L, and no threshold dose value was identified as safe for health.

**Conclusions:**

Results provide new evidence for risk assessment of low-medium concentrations of arsenic and contribute to the ongoing debate about the threshold-dose of effect, suggesting that even concentrations below 10 μg/L carry a mortality risk. Policy actions are urgently needed in areas exposed to arsenic like in the Viterbo province, to comply with current EU regulations.

## Introduction

Arsenic (As) is present in water predominately in its inorganic form that has been known to be associated with several chronic health consequences after life-long exposure, thus representing a major threat to human health. Arsenic has been classified as a human carcinogen group 1 based on consistent evidence of associations with lung, skin and bladder cancers, whereas limited evidence of carcinogenesis have been reported for liver, kidney and prostate cancer [1]. In addition, an association of arsenic exposure with cardiovascular, diabetogenic, respiratory, neurological, and developmental effects has been clearly depicted [2]. Most epidemiological evidence has come from populations chronically endemically exposed to very high arsenic levels in drinking water (>1000 μg/L) in Asian countries (Bangladesh, Taiwan, Vietnam, and India), in Argentina and Chile, and in several parts of the US (Arizona, California and Nevada) [3]. These studies showed an adverse effect of high As exposure in drinking water to specific diseases, while the risk related to low-medium exposure (<100 μg/L) is still not well characterized [4–9]. At these concentrations, the available evidence is insufficient to characterize the dose-response relationship and to identify a threshold-level for toxic arsenic effects [10]. At the same time, international agencies responsible for food and water safety consider arsenic toxic in any intake due to the multiplicity of ways of exposure [11], leaving as yet unresolved the debate to set a minimum standard value for human health.

To ensure that water can be consumed safely over a lifetime, the European Union in 1998 (EU) set a limit of 10 μg/L for arsenic concentrations in drinking water (DWD 98/83/EC) according to a WHO revision of the scientific knowledge [12]. Advocating the precautionary principle, some authors recently have called for a further lowering of the current standard [13].

Arsenic contamination of drinking water is a public health problem in several Italian areas due to the volcanic origins of the territory. Arsenic values in drinking water were chronically between 20 and 50 μg/L, in large areas of Italy (e.g. Toscana, Lombardia, Lazio, Campania), and since 2003 the Italian Government requested several derogations from the EU in order to allow structural interventions on the water supply system. However, in October 2010 the EU refused to yield a further derogation, and an official “state of emergency” for the water supply was declared in 128 Italian municipalities, 60 of which are located in Viterbo province, the northern part of the Lazio region. As result of the long derogation period, implementing mitigation measures was delayed for several years and the population did not modify their food or drinking water habits. Due to the peculiar hydrogeological characteristics of the Viterbo area [14, 15], it is likely that the local population has been exposed to arsenic at low-medium levels for a long time and it is possible that the actual intake is even higher than that considering the multiple sources of arsenic exposure (i.e. local foods). Since the beginning of the emergency, we carried out an ecological analysis to evaluate the health effects of arsenic exposure in drinking water measured at the municipal level; mortality excesses were found for several cancers and other chronic conditions such as cardiovascular and respiratory diseases in the municipalities of Viterbo province [16].

Considering these previous results, we planned a large population study, in which exposure was estimated at individual levels over a lifetime. The large sample size and the long study period considered in this study allows to analyse diseases with long latency, for which evidence on low-medium arsenic doses have been previously reported [4–9]. A further objective was to describe the shape of the dose-response relationship between lifetime exposure to arsenic and mortality risk for chronic diseases.

## Materials and Methods

### Area of the study

The area of Viterbo province is characterized by the presence of the Cimino-Vico volcanic system where a continuous basal aquifer flows within Pliocene-Pleistocene sedimentary rocks with a very high concentrations (up to 130–370 μg/L) of arsenic have been documented since the ‘70s [14, 15, 17–19]. This volcanic aquifer covers an area of 5,500 km^2^ and supplies water for human consumption (about 150,000 inhabitants) and local agricultural activities [15, 19]. The degree of water contamination is confirmed by the high content of bioavailable arsenic in the agricultural soil that reaches its highest levels in the Viterbo province [20]. The Viterbo area has a rural economy, air pollution levels are relatively low, and the others known health risks in the area are indoor radon exposure associated with lung cancer [21], pesticides used largely in agricultural settings [22] and occupational exposure to silica dust in the local traditional ceramic industry with health risks for silicosis [23] and lung cancer [24].

**Fig 1:**
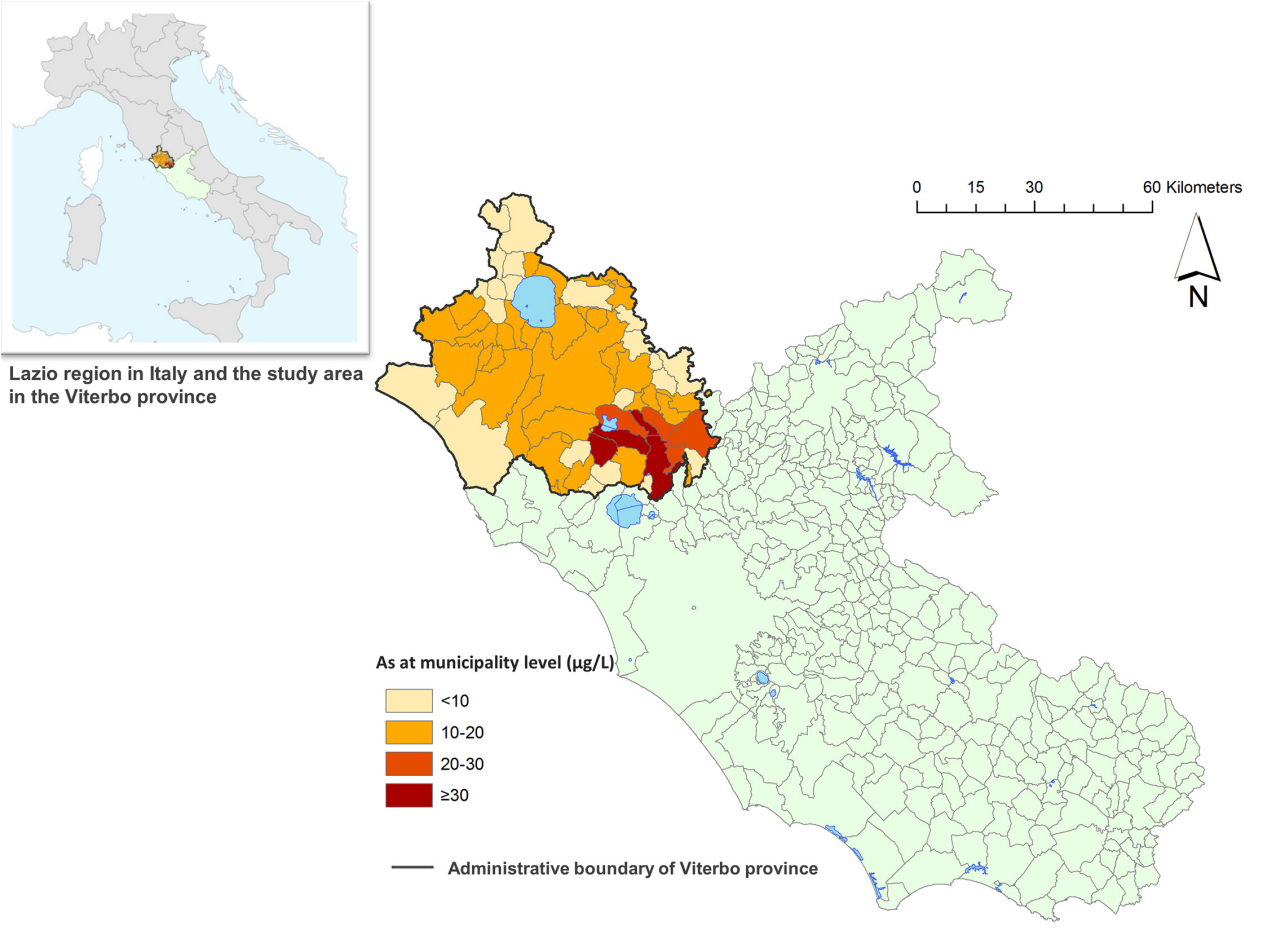
Mean concentration of arsenic in drinking water in the municipalities of Viterbo province in the Lazio Region, 1990–2010.

The location of Viterbo province in the Lazio Region and in Italy is illustrated in Fig 1.

### Study population

In the study area, a large administrative cohort of residents was enrolled selecting eight municipalities, with average arsenic at the municipal level ≥20 μg/L, and nine municipalities, with As ≤10 μg/L (Fig 1).

Subjects included in the study were all residents on January 1st 1990 and those who were subsequently born or immigrated to the municipality up to December 31th 2010. The data sources for *subjects enrollment* were population registries that provided address information for each resident at the beginning of the study period, and the first residential address for those registered later, after birth or immigration. For each subject, complete anagraphic history over lifetime was reconstructed from population archives, recording all changes of residency.

For each subject, *a mortality follow-up* was conducted through a record linkage with the Mortality Registry of the Lazio Region. *The beginning of the follow-up* was the date of birth or immigration to the study municipalities, and *the end* was December 31th, 2010. Each subject was censored at date of death, emigration or at the end of follow-up.

### Exposure estimates at the individual level

Arsenic levels in drinking water were derived from the database managed by the Environmental Protection Agency of the Lazio region (ARPA Lazio) [25]. In all Italian regions water for human consumption is measured for arsenic levels as mandated by law to monitor the quality of drinking water from every public water utility, and follows standardized procedures according to requirements fixed by the Italian Legislative Decree 31/2001 (transposition of the European Drinking Water Directive). The analytical determination of arsenic is performed by Atomic absorption spectrometry and Inductively Coupled Plasma Mass Spectrometry; both methods have a detection limit of ≤1 μg/L. Arsenic is monitored in specific sampling points within the municipality boundaries and a number of water samples are collected annually, proportional to population size, and equally distributed in time and location.

In Italy, municipal water supplies are managed by private companies or by the municipality itself. In the case of private companies, the municipal territory is divided into several supply units which deliver water directly to households. The map of these supply units were obtained from the local water companies within the study municipality.

ARPA Lazio data were not available for the entire study period, but only for 2005–2010. We assumed arsenic concentrations in the study area to be stable in the study period based on the widely known arsenic contamination in groundwater due to natural underlying geological processes [14, 15, 17–19] and to the absence of any arsenic mitigation intervention before 2010.

Individual arsenic exposure for each subject was defined using a spatial approach taking into account residential histories since birth, as suggested by other authors [26–29]. In Italy, all residences in an individual’s lifetime are recorded in a population registry that collects all demographic changes for every subject living or resident within a municipality, ensuring complete coverage and uniform quality level throughout the country.

In each municipality, we mapped the addresses of study subjects, the water supply unit connected with those addresses, and the addresses of As sampling points within the municipal territory using the ArcGIS software. To attribute a subject’s individual arsenic exposure, we used the following procedure:

- For subjects whose water was supplied by municipal aqueducts, all residential addresses were attributed arsenic concentrations by the Voronoi method [30] based on the nearest sampling points, (57%); for subjects whose water was supplied by a private company and residing within a specific water supply unit area arsenic concentration was determined by the “point-in-polygon” procedure [30] (33%). For 90% of subjects residential addresses were geocoded and an individual arsenic concentration was attributed.
- For the 10% of subjects residing in rural locations, we were not able to match addresses within the GIS, and the municipal average arsenic concentration was attributed [31].

For each subject two main indicators of individual exposure were calculated:

- an average of the individual arsenic exposure at the first year of residence (*As*_*I*_ in μg/l) [28]
- a cumulative arsenic dose indicator (*CAI* in μg) accounting for both intensity and duration of arsenic exposure over an individual’s lifetime, and for daily drinking water habits.

CAI indicator was calculated by multiplying arsenic concentrations from each subject’s residence by time lived at each address and by average water intake, summed up for all residencies since birth, using the formula below:

$$CAI=\sum_{i}{As}_{i}\times D_{i}\times Q$$

where *As*_*i*_ (in μg/l) and *D*_*i*_ (in person-days) are the estimated arsenic level and duration of residence at the i-th address and Q is average daily drinking water intake (*Q* = 0.8*L*/*day*) retrieved from the National Food Consumption Survey for the adult Italian population (18+ years old) [32]. Q was derived summing up the amount of drinking water and non-alcoholic beverages consumed (except milk-based beverages) such as coffee, tea, and fruit syrup diluted with tap water.

### Data analysis

We evaluated the effects of chronic arsenic exposure on mortality from several causes for which an association with low-medium arsenic exposure was suggested by previous studies (Table A in S1 File). The association between each arsenic exposure indicator and a specific mortality cause was investigated using a survival analysis through the Cox proportional hazard model [33].

We considered as potential confounders at individual level: age, calendar period, and employment in the ceramics industry (information linked with an occupational cohort) [23, 24]. Moreover socioeconomic position (SEP) at the census tract level calculated from the 2001 Census data information [34] was considered. The SEP index is a composite indicator based on five dimensions of socioeconomic deprivation (elementary education, unemployment, rental housing, mono-parental families, population density) [34]. The indicator is classified into quintiles from low (more deprived) to high (less deprived); the most deprived categories were characterized by overcrowding, low education level, unemployment, and non residence ownership and were associated with mortality excesses in all age groups and in both genders [34]. Radon exposure and smoking sales were available at the municipal level. Indoor radon exposure (Bq/m^3^) was retrieved from a monitoring campaign of about 3000 dwellings in Rome and Viterbo provinces from 2004 to 2008 by the National Environmental Protection Agency [35]. As_I_ was categorized as *As*_*I*_ ≤ 10*μg* / *L*, 10 < *As*_*I*_ < 20*μg* / *L* and *As*_*I*_ ≥ 20*μg* / *L* and CAI was categorized into quartiles: <25° percentile, 25°-75°percentile, >75° percentile (μ*g*). CAI exposure was included in the Cox model as a time-dependent variable. Age was considered as the time axis following the method suggested by other authors [36]; therefore, subjects entered at birth or their age of immigration and were censored at their age at the event (death) or censoring age (age of emigration or at 31^st^ December 2010 chosen as date of end of follow-up). Basically, subjects could have more than one exposure window, one for each residency, and they could contribute to more than one exposure category if they changed residency moving into a municipality with a different arsenic level. Time to event (in person-days) was therefore specific for a specific exposure category and accounted for the residential changes occurred during the study. Similarly, exposure duration (in years) was specific for each exposure category and was derived summing up the different time to event lived with that specific exposure level. All models were adjusted for potential confounders at individual level, other than age, entered as covariates. Since individual data were clustered within a municipality, to account for within-cluster correlations and between-cluster heterogeneity, a random intercept for the municipality variable was included in the models [37]. Basically, the random intercept is able to adjust for all characteristics that aggregate within a municipality, i.e. confounders measured at the municipal level (i.e. radon, smoking sales).

Proportional hazard assumption was examined through the Mantel-Cox test, and when the assumption was violated, stratified Cox models were performed.

Hazard Ratios (HR and 95% Confidence Intervals, 95%CI) were estimated considering as the reference category *As*_*I*_ < 10*μg* / *L* or the 25°percentile for As_I_ and CAI respectively. A test for trend was performed on all arsenic categories to assess if the association between exposure and study outcomes followed a dose-response trend with a linear component (at *p*-value<0.05) [38].

All models were stratified by gender. To account for a minimum latency period between exposure and death from chronic diseases, we studied only subjects who were resident for at least 5 years in the study municipalities. To check the robustness of the Cox model, a sensitivity analysis using a Poisson regression model was also performed.

As a sensitivity analysis, we used a third indicator as the exposure variable, the average daily water intake (*LDI* in μg/kg b.w./day) at the first residence. The *LDI* was calculated from *As*_*I*_, average daily drinking water intake for the adult population (*Q*), an age-specific average body weight (*BW* in kg), assuming 100% allocation to water in the chronic dietary exposure to arsenic through the formula

$$LDI={As}_{I}\times Q/BW\;.$$

Age-specific BW was available for the adult Italian population (18+ years old) from the National Food Consumption Survey [32]. LDI was categorized in quartiles.

### Evaluating the dose-response relationship

Associations between arsenic and the mortality outcomes found in the main analysis were explored more in depth by investigating dose-response relationships using a flexible non parametric approach in a Cox model framework [39]. A quadratic b-spline with 3 knots (at 10°, 50° and 90° percentile of the As_I_ distribution) was used for arsenic and models were adjusted for the same confounding variables used in the main analysis. Sensitivity analysis was run using different splines and different combinations of knots.

To assess the hypothesis of linearity of dose-response curves, we compared the beta coefficients for step increases in As_I_ exposure. The analysis was run using the *dlnm* and *survival* packages in R 3.0.2

### Ethics statements

This study is based on routine administrative data that our Department, as a public institution, is authorized by the Regional Health Service to use for epidemiological purposes without formal approval from an Ethics Committee. Administrative records were made anonymous and de-identified prior to analysis, and no informed consent was needed.

## Results

**Fig 2:**
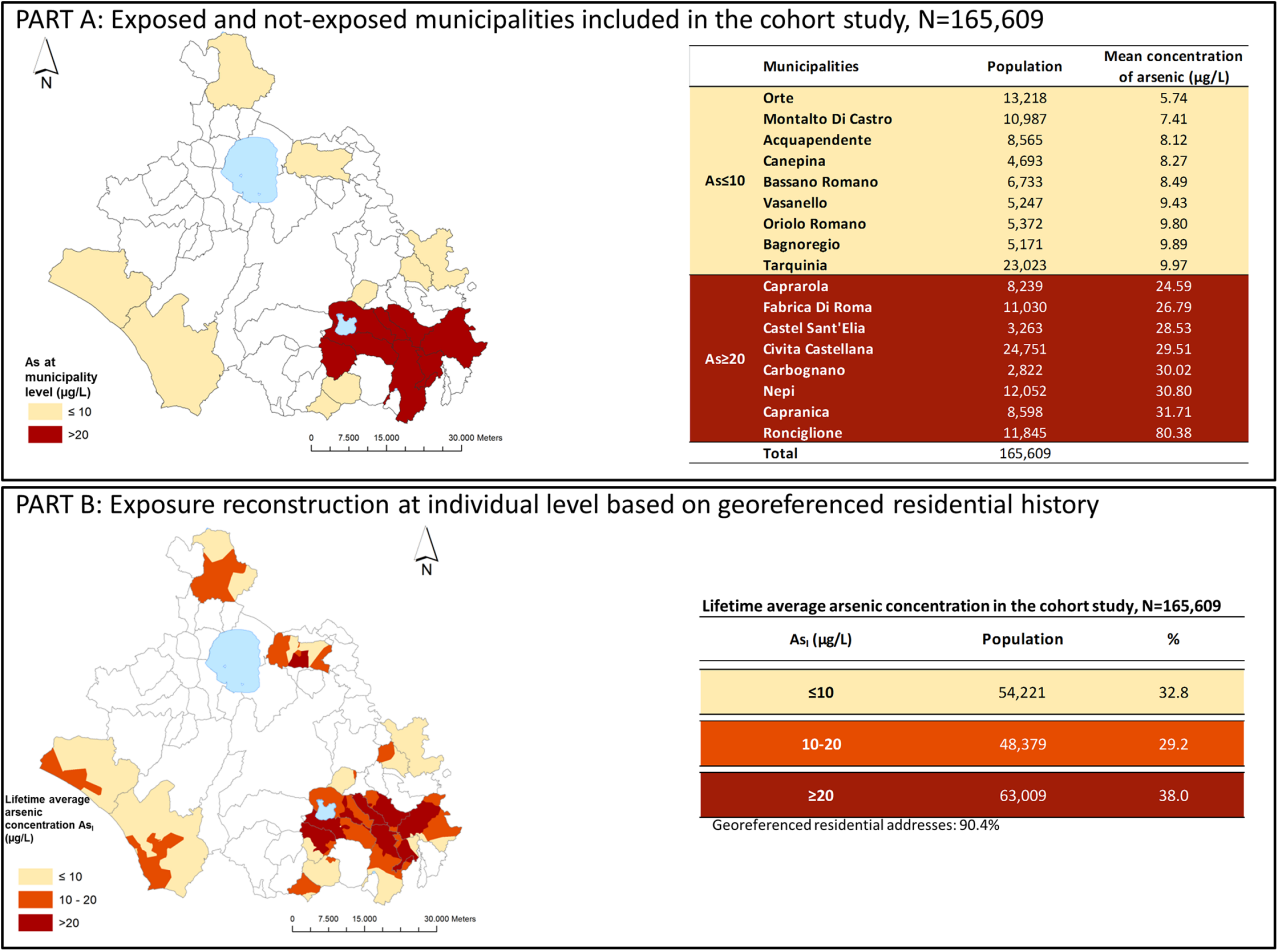
Exposure assessment within the municipalities included in the study, 1990–2010.

The study population included 165,609 subjects for whom individual exposure was attributed. Fig 2 shows arsenic concentrations in the study area.

Part A shows the resident population of the 17 municipalities included in the study by average municipal arsenic levels. In the study period, 755 water samples were collected, (13% of which from municipal wells), corresponding to 10 samples per year on average.

Results from the exposure reconstruction at individual level by spatial analysis are reported in Part B. For 90.4% of subjects As_I_ exposure was successfully determined from geocoded residential addresses. For the remaining subjects, As_I_ was attributed from average municipal exposure. As_I_ exposure ranged between 0.5 μg/L (1° pctile) and 80.4 μg/L (99° pctile) with a mean value of 19.3 μg/L but 38% of subjects had been exposed to As_I_ >20 μg/L. In the ≤10, 10–20 μg/L and ≥20 μg/L exposure categories the mean (standard deviation) As_I_ is 6.5 μg/L (SD = 2.8 μg/L), 13.7 μg/L (SD = 2.6 μg/L) and 34.5 μg/L (SD = 19.7 μg/L) respectively (Fig A in S1 File). The mean CAI value was 230.9 μg ranging from 0.7 μg (1^st^ percentile) to 1.6 g (99^th^ percentile).

The characteristics of the population under study are reported in Table 1. The mean age of the study population was 32 years and the proportion of deaths that occurred before the end of the study was 12.5% with a mean age at death of 66 years, with a higher proportion of ≥65 years subjects in the category As_I_ <10 μg/l. The proportion of deaths was higher in the most exposed category (13.6%). Duration of residence was longer than 20 years for almost 50% of the residents and for more than 95% of subjects who died. Average duration of exposure was 39.5 years and 69% of subjects had been exposed longer than 20 years (97% among subjects who died). The proportion of subjects classified at medium-low/low socioeconomic level tends to increase with greater As_I_ exposure (*p-*value<0.001). A similar pattern was observed for average radon exposure and for the silica dust exposure from the occupational histories (*p*-value<0.001).

**Table 1:** Characteristics of the study population and deaths by individual arsenic exposure (As_I_), 1990–2010.

	Study population (n = 165,609)				Deaths (n = 20,776)			
	Arsenic exposure categories				Arsenic exposure categories			
	≤ 10 (n = 54,221)	10–20 (n = 48,379)	>20 (n = 63,009)		≤10 (n = 6,891)	10–20 (n = 5,317)	>20 (n = 8,568)	
	n (%)	n (%)	n (%)	*p*-value	n (%)	n (%)	n (%)	*p*-value
**Gender**								
Male	26938 (49.7)	23972 (49.6)	31259 (49.6)		3553 (51.6)	2759 (51.9)	4502 (52.5)	
Female	27283 (50.3)	24407 (50.4)	31750 (50.4)	0.818	3338 (48.4)	2558 (48.1)	4066 (47.5)	0.218
**Georeferenced residential addresses**	48198 (88.9)	47041 (97.2)	54414 (86.4)					
**Age at enrollment (years)**								
mean ± SD	32.8 ± 23.5	31.9 ± 23.3	31.5 ± 23.7		66.4 ± 13.1	66.2 ± 13.2	65.6 ± 13.6	
≤35	30840 (56.9)	28032 (57.9)	37404 (59.4)		182 (2.6)	145 (2.7)	277 (3.2)	
36–64	17149 (31.6)	15341 (31.7)	18545 (29.4)		2428 (35.2)	1963 (36.9)	3194 (37.3)	
>65	6230 (11.5)	5006 (10.3)	7055 (11.2)	0.174	4281 (62.1)	3209 (60.4)	5097 (59.5)	<0.001
**Residence duration as of 1st January 1990 (years) **								
≤ 5	20513 (37.8)	17713 (36.6)	22228 (35.3)		199 (2.9)	224 (4.2)	122 (1.4)	
5–10	2328 (4.3)	2085 (4.3)	2822 (4.5)		52 (0.8)	45 (0.8)	31 (0.4)	
10–20	5702 (10.5)	5283 (10.9)	6960 (11)		118 (1.7)	61 (1.1)	84 (1.0)	
> 20	25678 (47.4)	23298 (48.2)	30999 (49.2)	<0.001	6522 (94.6)	4987 (93.8)	8331 (97.2)	<0.001
**Exposure duration as of 31th December 2010 (years) **								
≤ 5	9877 (18.2)	7448 (15.4)	9484 (15.1)					
5–10	3064 (5.7)	3051 (6.3)	3613 (5.7)		84 (1.2)	100 (1.9)	61 (0.7)	
10–20	4623 (8.5)	4428 (9.2)	6157 (9.8)		137 (2)	142 (2.7)	90 (1.1)	
> 20	36657 (67.6)	33452 (69.1)	43755 (69.4)	<0.001	6670 (96.8)	5075 (95.4)	8417 (98.2)	<0.001
**Socioeconomic position**								
High	6986 (12.9)	10543 (21.8)	7915 (12.6)		581 (8.4)	895 (16.8)	561 (6.5)	
medium-high	13854 (25.6)	10184 (21.1)	16375 (26.0)		953 (13.8)	1300 (24.4)	1248 (14.6)	
Medium	10430 (19.2)	11165 (23.1)	11620 (18.4)		926 (13.4)	1082 (20.3)	1117 (13.0)	
medium-low	7151 (13.2)	10002 (20.7)	9225 (14.6)	<0.001	623 (9.0)	817 (15.4)	684 (8.0)	<0.001
Low	15800 (29.1)	6485 (13.4)	17874 (28.4)		3808 (55.3)	1223 (23.0)	4958 (57.9)	
**Radon (Bq/m** ^**3**^ **)**								
≤200	48862 (90.1)	29639 (31.3)	27050 (42.9)		6555 (95.1)	3830 (72.0)	3517 (41.0)	
>200	5359 (9.9)	18740 (38.7)	35959 (57.1)	<0.001	336 (4.9)	1487 (28.0)	5051 (59.0)	<0.001
**Cigarette consumption per capita (per year)**								
≤1200	34223 (63.1)	18055 (37.3)	13440 (21.3)		4861 (70.5)	2465 (46.4)	1556 (18.2)	
>1200	19998 (36.9)	30324 (62.7)	49569 (78.7)	<0.001	2030 (29.5)	2852 (53.6)	7012 (81.8)	<0.001

### Health effects from arsenic concentration at the individual level (As_I_)

We analysed 138,800 subjects after excluding 26,809 subjects who resided in the area for fewer than 5 years. Results of the survival analysis for As_I_ are shown in Table 2. Mortality risks from all natural causes increased with higher As_I_ exposure in both genders, with positive trends found for most causes; risks tended to be higher in males (10–20 μg/L: HR = 1.27, >20 μg/L: HR = 1.51) than in females (10–20 μg/L: HR = 1.14, >20 μg/L: HR = 1.19).

**Table 2:** Associations of average arsenic exposure at first year of residence (As_I_) and mortality for specific causes (HR, 95% Confidence Intervals, 95% CI) in the study, 1990–2010.

	As_I_ (μg/L)					
Causes of death (ICD-9)	≤10^a^		10–20^a^		> 20^a^	*p*-value for trend
	Deaths	Deaths	HR^b^ ^,^ ^c^ (95% CI)	Deaths	HR^a^ ^,^ ^b^ (95% CI)	
**Males (N = 68758)**						
Natural causes (001–799)	3553	2759	**1.27 (1.18, 1.38)**	4502	**1.51 (1.36, 1.67)**	**<0.001**
Malignant cancers (140–208)	1068	841	**1.27 (1.11, 1.45)**	1451	**1.51 (1.28, 1.78)**	**<0.001**
Liver and bile ducts (155, 156)	64	45	1.20 (0.78, 1.87)	98	**1.58 (1.08, 2.33)**	**0.019**
Tracheas, bronchus and lung (162)	283	259	**1.47 (1.17, 1.86)**	469	**1.83 (1.41, 2.39)**	**<0.001**
Prostate (185)	89	60	1.21 (0.86, 1.72)	98	1.15 (0.86, 1.53)	0.360
Bladder (188)	54	32	0.95 (0.56, 1.62)	71	1.36 (0.82, 2.25)	0.229
Kidney (189)	24	18	1.45 (0.72, 2.93)	26	1.22 (0.64, 2.33)	0.546
Circulatory system diseases (390–459)	1317	1061	**1.39 (1.23, 1.58)**	1659	**1.54 (1.32, 1.80)**	**<0.001**
Ischemic heart disease (410–414)	380	310	**1.42 (1.15, 1.75)**	567	**1.70 (1.33, 2.16)**	**<0.001**
Myocardial infarction (410)	202	139	**1.32 (0.99, 1.75)**	302	**1.74 (1.30, 2.33)**	**<0.001**
Coronary atherosclerosis (414)	168	162	**1.50 (1.11, 2.01)**	248	**1.58 (1.14, 2.19)**	**0.008**
Cerebrovascular diseases (430–438)	372	263	**1.44 (1.14, 1.80)**	443	**1.50 (1.16, 1.94)**	**0.002**
Stroke (430, 431, 434, 436)	301	211	**1.47 (1.14, 1.90)**	365	**1.59 (1.20, 2.12)**	**0.001**
Peripheral Arterial (440–448)	93	101	**1.52 (1.03, 2.25)**	102	1.26 (0.79, 2.01)	0.230
Respiratory system diseases (460–519)	190	163	**1.48 (1.09, 2.01)**	393	**1.94 (1.38, 2.72)**	**<0.001**
COPD (490–496)	78	81	**1.84 (1.24, 2.72)**	160	**2.04 (1.41, 2.96)**	**<0.001**
Diabetes mellitus (250)	67	46	1.25 (0.78, 2.01)	110	1.43 (0.91, 2.23)	0.122
**Females (N = 70042)**						
Natural causes (001–799)	3338	2558	**1.14 (1.05, 1.24)**	4066	**1.19 (1.07, 1.33)**	**0.001**
Malignant cancers (140–208)	717	648	**1.26 (1.08, 1.47)**	961	**1.32 (1.08, 1.60)**	**0.004**
Liver and bile ducts (155, 156)	45	46	1.57 (0.80, 3.06)	65	1.45 (0.62, 3.41)	0.398
Tracheas, bronchus and lung (162)	63	69	**1.80 (1.23, 2.66)**	100	**1.69 (1.18, 2.42)**	**0.015**
Bladder (188)	18	11	1.19 (0.50, 2.82)	9	0.57 (0.23, 1.36)	0.292
Kidney (189)	10	11	2.17 (0.84, 5.59)	10	1.09 (0.43, 2.74)	0.871
Circulatory system diseases (390–459)	1435	1054	**1.17 (1.03, 1.33)**	1855	**1.20 (1.03, 1.40)**	**0.020**
Ischemic heart disease (410–414)	304	263	**1.36 (1.06, 1.74)**	447	1.23 (0.92, 1.65)	0.171
Myocardial infarction (410)	121	104	**1.43 (1.00, 2.04)**	172	1.38 (0.94, 2.03)	0.100
Coronary atherosclerosis (414)	180	156	**1.43 (1.05, 1.95)**	267	1.27 (0.91, 1.76)	0.199
Cerebrovascular diseases (430–438)	454	279	1.17 (0.95, 1.43)	530	**1.26 (1.02, 1.56)**	**0.030**
Stroke (430, 431, 434, 436)	359	237	**1.23 (0.99, 1.52)**	424	**1.28 (1.03, 1.59)**	**0.026**
Peripheral Arterial (440–448)	89	91	1.20 (0.79, 1.83)	104	1.24 (0.72, 2.14)	0.391
Respiratory system diseases (460–519)	127	107	**1.51 (1.05, 2.16)**	185	1.26 (0.86, 1.85)	0.253
COPD (490–496)	46	42	1.59 (0.96, 2.65)	69	1.49 (0.93, 2.38)	0.152
Diabetes mellitus (250)	95	84	**2.12 (1.45, 3.11)**	200	**2.08 (1.47, 2.94)**	**<0.001**

^a^ Person time of exposure of individual study subjects in the three As_I_ exposure categories: Males: As_I_ ≤10 μg/L: n = 379,421; As_I_ = 10–20 μg/L: n = 350,192; As_I_≥20 μg/L: n = 442,993; Females: As_I_ ≤10 μg/L: n = 392,439; As_I_ = 10–20 μg/L: n = 363,084; As_I_≥20 μg/L: n = 461,136

^b^ HR: Hazard Ratios and 95%CI calculated respect to As≤10 μg/L as reference group; significant HR and p-values for trend highlighted in bold

^c^ Models adjusted for age, calendar period, socioeconomic level, occupation in the ceramic industry, smoking sales and radon exposure

Significant excesses were found for the entire group of malignant cancers (>20 μg/L: HR = 1.51 in males and HR = 1.32 in females), and for lung cancer (>20 μg/L: HR = 1.83 in males and HR = 1.69 in females). For liver cancer, an excess was found only in males (>20 μg/L: HR = 1.58), while no excesses were observed for prostate cancer or for bladder and kidney cancers in either gender.

We observed significant excesses for the entire cardiovascular group (>20 μg/L: HR = 1.54 in males and HR = 1.20 in females), with higher risks in males for ischemic heart diseases (>20 μg/L: HR = 1.70) and in females for stroke (>20 μg/L: HR = 1.28). Positive trends were observed for all cardiovascular diseases except peripheral arterial disease in males and for cerebrovascular diseases in females. For peripheral arterial diseases in men and for ischemic heart diseases in women, a mortality excess was found only in the 10–20 μg/L category.

Respiratory mortality in males was increased in the two exposure categories, whereas for females was higher only in the 10–20 μg/L exposure group. In males, excesses for COPD were also found (>20 μg/L: HR = 2.04).

For diabetes, only females showed a significant excess risk in both exposure categories (10–20 μg/L: HR = 2.12, >20 μg/L: HR = 2.08).

The sensitivity analysis with minimally adjusted models revealed robust results for the >20 μg/L category but not for the 10–20 μg/L group (Table D in S1 File).

### Health effects from cumulative arsenic doses (CAI)

For most diseases, mortality excesses for CAI are higher than those estimated for As_I_ and confirm that the highest risks are in males (Table 3). Among malignant cancers excess risk was found for lung cancer in males (25–75° pctile: HR = 2.03, >75° pctile: HR = 2.61, *p*-value<0.001) and, in females only in the highest exposure category (>75° pctile: HR = 2.09, *p*-value = 0.014). Results of circulatory disease overlap findings from the analysis for *As*_*I*,_ showing increasing risks with higher exposures. Risks were higher for myocardial infarction in males (25–75° pctile: HR = 1.90, >75° pctile: HR = 2.94) and for cerebrovascular disease in females (25–75° pctile: HR = 1.69, >75° pctile: HR = 1.87). The CAI analysis confirms an effect on respiratory causes and COPD in males (25–75° pctile: HR = 2.20, >75° pctile: HR = 2.54) and on diabetes in females in the highest category (>75° pctile: HR = 2.56, *p*-value<0.001).

**Table 3:** Associations of individual cumulative arsenic dose (CAI) and mortality from specific causes (HR, 95% Confidence Intervals, 95% CI) in the study, 1990–2010.

	CAI (μg)					
Causes of death (ICD-9)	(≤ 204.9 μg)^a^		(204.9–804.0 μg)^a^		(>804.0 μg)^a^	*p*-value for trend
	Deaths	Deaths	HR^b^ ^,^ ^c^ (95% CI)	Deaths	HR^a^ ^,^ ^b^ (95% CI)	
**Males (N = 68758)**						
Natural causes (001–799)	748	4589	**1.59 (1.45, 1.74)**	5477	**2.02 (1.82, 2.25)**	**<0.001**
Malignant cancers (140–208)	201	1459	**1.69 (1.43, 2.00)**	1700	**2.17 (1.79, 2.62)**	**<0.001**
Liver and bile ducts (155, 156)	14	87	1.31 (0.72, 2.37)	106	1.62 (0.88, 3.00)	0.089
Tracheas, bronchus and lung (162)	53	419	**2.03 (1.48, 2.79)**	539	**2.61 (1.84, 3.71)**	**<0.001**
Prostate (185)	17	105	1.19 (0.71, 1.99)	125	1.32 (0.79, 2.20)	0.239
Bladder (188)	13	56	0.82 (0.43, 1.58)	88	1.32 (0.67, 2.60)	0.114
Kidney (189)	4	34	1.95 (0.67, 5.72)	30	1.93 (0.63, 5.96)	0.418
Circulatory system diseases (390–459)	242	1720	**1.61 (1.38, 1.87)**	2075	**2.06 (1.73, 2.46)**	**<0.001**
Ischemic heart disease (410–414)	64	519	**1.87 (1.41, 2.51)**	674	**2.61 (1.90, 3.60)**	**<0.001**
Myocardial infarction (410)	33	266	**1.90 (1.28, 2.82)**	344	**2.94 (1.92, 4.51)**	**<0.001**
Coronary atherosclerosis (414)	29	238	**1.78 (1.16, 2.72)**	311	**2.10 (1.33, 3.33)**	**0.003**
Cerebrovascular diseases (430–438)	65	471	**1.50 (1.12, 2.02)**	542	**1.72 (1.24, 2.39)**	**0.003**
Stroke (430, 431, 434, 436)	56	373	**1.42 (1.03, 1.97)**	448	**1.74 (1.22, 2.48)**	**0.002**
Peripheral Arterial (440–448)	14	133	**2.20 (1.20, 4.04)**	149	**2.44 (1.27, 4.66)**	**0.024**
Respiratory system diseases (460–519)	35	260	**1.53 (1.03, 2.27)**	451	**1.76 (1.14, 2.71)**	**0.017**
COPD (490–496)	11	121	**2.20 (1.13, 4.30)**	187	**2.54 (1.27, 5.08)**	**0.016**
Diabetes mellitus (250)	15	87	1.25 (0.68, 2.29)	121	1.30 (0.67, 2.50)	0.482
**Females (N = 70042)**						
Natural causes (001–799)	720	4021	**1.45 (1.32, 1.58)**	5221	**1.59 (1.43, 1.77)**	**<0.001**
Malignant cancers (140–208)	147	1009	**1.73 (1.43, 2.11)**	1170	**2.08 (1.67, 2.60)**	**<0.001**
Liver and bile ducts (155, 156)	11	68	1.32 (0.63, 2.74)	77	1.18 (0.50, 2.77)	0.821
Tracheas, bronchus and lung (162)	15	98	1.66 (0.93, 2.95)	119	**2.09 (1.15, 3.80)**	**0.014**
Bladder (188)	5	18	0.91 (0.31, 2.67)	15	0.71 (0.23, 2.24)	0.482
Kidney (189)	1	18	4.51 (0.58, 35.3)	12	3.03 (0.37, 25.22)	0.803
Circulatory system diseases (390–459)	241	1732	**1.65 (1.41, 1.92)**	2371	**1.70 (1.42, 2.02)**	**<0.001**
Ischemic heart disease (410–414)	57	394	**1.57 (1.15, 2.15)**	563	**1.60 (1.13, 2.26)**	**0.036**
Myocardial infarction (410)	23	170	1.54 (0.95, 2.49)	204	1.50 (0.89, 2.53)	0.277
Coronary atherosclerosis (414)	34	219	**1.52 (1.01, 2.28)**	350	**1.69 (1.09, 2.61)**	**0.032**
Cerebrovascular diseases (430–438)	70	529	**1.69 (1.28, 2.23)**	664	**1.87 (1.38, 2.53)**	**0.001**
Stroke (430, 431, 434, 436)	60	417	**1.58 (1.17, 2.13)**	543	**1.82 (1.32, 2.51)**	**0.001**
Peripheral Arterial (440–448)	20	105	1.32 (0.78, 2.26)	159	1.38 (0.78, 2.45)	0.345
Respiratory system diseases (460–519)	31	150	1.00 (0.64, 1.57)	238	1.11 (0.68, 1.79)	0.573
COPD (490–496)	10	54	0.96 (0.46, 2.01)	93	1.30 (0.62, 2.75)	0.220
Diabetes mellitus (250)	17	120	1.43 (0.82, 2.52)	242	**2.56 (1.43, 4.57)**	**<0.001**

^a^ Person time of exposure of individual study subjects in the three CAI exposure categories: Males: CAI≤ 204.9 μg: n = 296,778; CAI = 204.9–804.0 μg: n = 595,344; CAI>804.0 μg: n = 282,942; Females: CAI≤ 204.9 μg: n = 294,332; CAI = 204.9–804.0 μg: n = 607,538; CAI>804.0 μg: n = 317,308

^b^ HR: Hazard Ratios and 95%CI calculated respect to CAI ≤25° pct (204.9 μg) as reference group; significant HR and p-values for trend highlighted in bold

^c^ Models adjusted for age, calendar period, socioeconomic level, occupation in the ceramic industry, smoking sales and radon exposure

The sensitivity analysis with minimally adjusted models revealed robust results for both CAI exposure categories (Table D in S1 File).

Results from the sensitivity analyses for LDI indicator are reported in Table B in S1 File and showed similar excesses to those found for CAI. In addition, the LDI indicator revealed a mortality excess for liver cancer in both genders (>75° pctile: HR = 2.05 in males; 25–75° pctile: HR = 2.03, >75° pctile: HR = 2.88 in females), for bladder cancer in males in the highest exposure category (>75° pctile: HR = 3.35, p-value = 0.002), and for kidney cancer in females in the highest exposure category, with borderline significance (>75° pctile: HR = 3.64, p-value = 0.069). In addition, significant associations were also found for respiratory diseases (25–75° pctile: HR = 1.50, >75° pctile: HR = 1.71), and diabetes mellitus in females (25–75° pctile: HR = 1.92, >75° pctile: HR = 2.38).

The sensitivity analysis using Poisson regression provided similar results to the Cox analysis.

### Dose-response relationship

**Fig 3:**
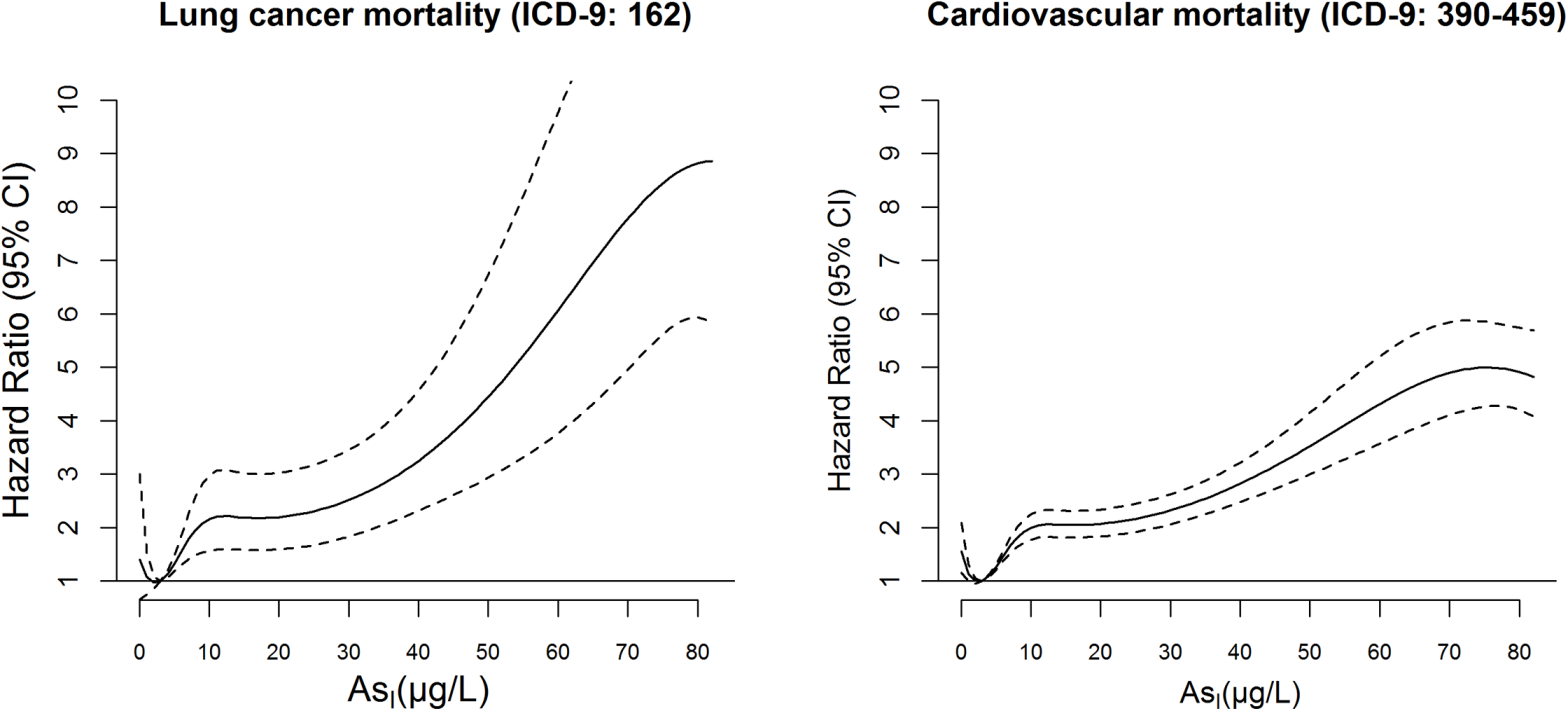
Dose-response relationship between lifetime average arsenic concentrations at the individual level (As_I_) and mortality risk for lung cancer and cardiovascular diseases (HR, 95% Confidence Intervals, 95% CI) in the study subjects, 1990–2010.

Fig 3 shows the dose-response curves for mortality from lung cancer and total cardiovascular causes, for which the main analysis revealed consistent associations.

A visual inspection of the curve for lung cancer shows different linear pieces, with similarly steep slope below 10 μg/L and above 30 μg/L while for cardiovascular diseases the curve is steeper at lower doses and then looks like more flat, and in both cases no threshold level could be identified as safe for health.

In the sensitivity analysis, the choice of different functions (natural spline, polynomial function or a cubic B-spline) did not change the shape of the relationship. The functions were sensitive to the knot’s selection, especially for those placed within the first decile of arsenic distribution, and to the number of knots, being lowered the effect at low doses when reducing the number of knots. Estimates of beta coefficients for subsequent 10 μg/L step increases in As_I_ exposure confirm the hypothesis of piecewise linear functions with different slopes (Table C in S1 File). Similar dose-response curves were observed for other mortality causes (i.e. diabetes, respiratory causes) although the relationship was less clear due to the small numbers.

## Discussion

This is one of the largest studies conducted in Europe to evaluate the health effects of arsenic in drinking water in an area with concentrations within a low-medium range (1 to 80 μg/L), on a population with long-term exposure (40 years on average).

The large study size allowed arsenic effects to be evaluated on a variety of different mortality outcomes, and the population-based administrative cohort approach with a GIS methodology, consistent with other studies [26–29], was a feasible approach to attribute lifetime arsenic exposure at the individual level, taking into account duration of residence. The long follow-up period allowed the evaluation of lifetime risks for long-latency diseases [40], adding more value than other similar studies on low-medium exposed areas have [4–8, 27–29, 41–43].

Our findings provide new evidence that even at these levels, arsenic is associated with mortality from several chronic conditions such as lung cancer, diseases of the circulatory system, respiratory diseases and diabetes. The associations are strong and risks become over twice the reference when we used the CAI indicator, which accounted for both exposure intensity and duration and are of similar magnitude of those observed in endemic countries as Taiwan at higher doses [44]. Previous studies of other areas with low-medium arsenic levels, based on environmental arsenic measures [45] or biospecimens [46–49], provided risks from chronic diseases similar to ours for magnitude of effect estimates; while few studies have reported no significant associations [27, 50–52].

The plausibility of these findings is supported by the positive dose-response relationship between mortality risks and arsenic exposure that we found for lung cancer and total cardiovascular causes. Similar results were described in a smaller cohort enrolled in the Strong Heart study in the US [45, 46], and in an ecological study in Spain on arsenic effects cardiovascular mortality [41]. In addition, the dose-response analysis shows an effect even for concentrations less than 10 μg/L. These findings make an important contribution to the debate about the threshold-dose of arsenic, providing evidence that even arsenic concentrations below the EU limit (10 μg/L) can raise mortality risks [10, 13].

An important limitation of the analysis is the incomplete adjustment for some individual risk factors like smoking habits, BMI and other lifestyle habits; in our study a socioeconomic indicator, at the census tract level, was a proxy for lifestyle habits [34]. The lack of individual data on these important covariates could have biased the effect estimates, especially for pathologies like lung cancer and cardiovascular diseases for which lifestyle habits are key risk factors and, as regards smoking habits we could only control for smoking information at the municipal level derived from cigarette sales. In our study, the large population size made it unfeasible to gather individual information on lifestyle habits.

We found a consistent association between arsenic and lung cancer. Recently, a cohort analysis in another non-endemic country observed effects similar to ours [42], while a previous low-dose meta-analysis found borderline significant effects for this cancer type [4, 6]. This finding, considering its biological plausibility, is coherent with IARC evaluation regarding the carcinogenic role of arsenic on lung cancer [1]. Its effect on lung cancer is robust to adjustment for other important risk factors for the disease, such as occupation in the ceramics industry, radon exposure, and tobacco sales at the municipal level that allow to control only some of the confounding due to smoking.

Among the other malignant cancers, we detected associations for liver and, less strongly, for kidney cancers for which there is limited epidemiological evidence in both high and low dose studies [1], while a cohort study carried out in non-endemic areas did not find any effect on these cancers [45]. In our study, an indication of arsenic’s effect on bladder cancer was found only in the daily intake indicator and the inconsistent findings could partly be explained by the use of mortality data since bladder cancer has a good survival especially in countries like Italy with good health care systems. In fact, in the study area the background-lifetime mortality risk for this disease is low, being 0.1% in females and 0.6% in males with over 40 years of exposure [53].

A strong association was also found for cardiovascular diseases, specifically for myocardial infarction in males and for stroke in females. A meta-analysis of low-to-moderate dose studies provided only a borderline association [8] while a recent study from the US suggested more clearly an arsenic effect on coronary heart disease and stroke [46]. Supporting of our results, a number of recent epidemiological studies have provided evidence on preclinical cardiac damage outcomes, such as carotid plaque, increased intima media thickness, endothelial dysfunction and vascular inflammation in populations exposed to moderate arsenic concentrations [8, 9, 46]. Experimental studies are helping to clarify pathogenic mechanisms [9].

An association of arsenic with mortality from diabetes was found and this is coherent with a cohort study on a non-endemic population [44], and few others on objective diabetes measures (i.e. fasting blood glucose levels), while other studies of low dose exposure provided inconsistent evidence [7]. In our study, this effect is restricted to women, similar to what already was found in the US by James et al. [44]. This greater susceptibility of women could be due to more severe clinical consequences of diabetes as suggested by Chiou et al. [54] in Taiwan, where they found a higher prevalence of vascular diseases associated with diabetes in females than in males.

We found an effect of arsenic on respiratory causes and, specifically, on COPD with more consistent associations in males. No direct comparisons can be made with other studies because they considered non-fatal outcomes, such as prevalence of respiratory symptoms or lung function [55–58]. These results have to be interpreted carefully due to the lack of a clear evidence on the mechanisms of arsenic on these end points. One hypothesis is that arsenic is linked to a decreased immune response to infections due to altered expression of cytokines genes and their receptors [59]. Other possible mechanisms consist of the depositing of arsenic on the lung epithelium causing tissue inflammation and consequent pulmonary fibrosis and lung function impairment or in arsenic-mediated damage to the Clara cells of the alveolar epithelium, leading to reduced secretion of the anti-inflammatory CC16 protein [60].

For most of the outcomes considered we found more consistent and stronger effects of arsenic on males, similar to previous studies [61, 62]. The greater susceptibility in males could be explained by hormonal and other biological differences, as suggested by animal studies that have showed that arsenic interacts with steroid hormones [63–66]. Also, methylation efficiency might differ between males and females residents, as suggested by a small biomonitoring study carried out previously in the same area [67].

A limitation of our exposure assessment was the lack of measurements in water utilities before 2005. Our assumption of constant As levels throughout the study period is supported by geological data which document stable arsenic contamination of the hydrothermal system that has supplied drinking water in the study area since the ‘70s [14, 15, 17–19] and to the lack of any remediation treatment in that time due to the long period of derogations [68]. In a sensitivity analysis, the intraclass correlation coefficient between the repeated arsenic measures within each municipality demonstrates consistency across years 2005–2010, in support of our assumption of the constancy of exposure over time (consistency = 0.80).

In the study we considered only the exposure due to arsenic from drinking water, and we cannot exclude that we underestimated the real population exposure due to all potential arsenic sources other than drinking water. Studies on spot samples in local wells in the area reporting As levels up to 240 μg/L [67, 69], and studies on phytoavailable arsenic in local agricultural soil showing high concentration in wheat (55.0 ng g^-1^ of dw) [20], confirm additional population exposure to inorganic arsenic due to local agriculture-based products, given that the area is predominantly a rural economy. Moreover, although our LDI indicator (average 0.27 μg/kg body weight per day) was based on the intake only from water, it falls within the range of the highest dietary exposure (95th percentile) to inorganic arsenic in the European populations (0.14 to 0.64 μg/kg b.w.per day) which considered all foods and beverages consumption [70]. The daily drinking water rate (0.8 liters/day) considered in the calculation of LDI and CAI derived from a survey on food consumption habits on a representative sample of 3000 Italian adults using internationally harmonized instruments (i.e. food diaries, family and individual questionnaires) [32] and being part of the exposure assessment at European level undergone by EFSA [11, 70], and that can have lead only to an underestimate of the actual intake of arsenic and, therefore, of the estimated risks in our study.

In the present study, relative risk estimates of arsenic effects cannot be directly interpreted into attributable risks [71], mainly due to the lack of control for the other multiple risk factors for the study outcomes (i.e. active smoking, BMI, lifestyles). Despite this limitation, hazard ratios for the category As_I_ >20 μg/L and for CAI indicator could be informative for risk assessment evaluations, since they are robust to the control for the set of confounders available in the study and to sensitivity analysis. Estimates for the As_I_ 10–20 μg/L category are inconsistent across the different endpoints and confounded by socioeconomic position, moreover there is a poor contrast in terms of exposure distribution (median As_I_: 13.0 *vs* 7.4 μg/L) respect to the reference group. Regulatory bodies (EPA, WHO) call for more epidemiological research on non-endemic areas to inform risk assessment even at low-medium doses [39, 72]. In the United States, estimates of excess mortality for cancers (lung, bladder and liver) are on the order of magnitude of 5 per 100,000 in the population, being approximately 5% the fraction of exposed to arsenic >20 μg/L [72]. In Europe risk assessment estimates are lacking, but in Italy the population exposed to arsenic level >10 μg/L can be estimated as 1.7% [73], much lower than in our study, that have to be taken into account in risk assessment exercise for the general population [72].

In our study no direct arsenic measures from biomarkers or estimated from questionnaires were available, but these methods are unfeasible in such a large study and are not informative beyond the previous few months [74]. A small biomonitoring study in the same area [65], found levels of urinary inorganic arsenic (75° pctile 1.89 μg/L) above the reference range in the Italian population (0.1–1.5 μg/L) defined from the Italian Society of Reference Values (http://www.valoridiriferimento.it/),.

## Conclusion

In our study lifetime arsenic exposure from drinking water at low-medium levels was associated with mortality risk from lung cancer, cardiovascular diseases, COPD and diabetes.

Meanwhile the debate over threshold-dose of health risks from arsenic is ongoing [10, 13], our large study provides new evidence for risk assessment among populations chronically exposed to low-medium doses over lifetime as in Viterbo province, and underlines the need for actions to comply with the current regulatory standard of 10 μg/L.

## Supporting Information

S1 File Table A. Health status indicators included in the study, related evidence from epidemiological studies on populations exposed to low-moderate arsenic doses (<100 μg/L) in drinking water and possible underlying mechanisms. TableB. Associations of individual daily intake (LDI) and mortality from specific causes (HR, 95% Confidence Intervals, 95% CI) in the study, 1990–2010. Table C. Effects of average arsenic concentrations at first year of residence (As_I_) and mortality for specific causes (beta coefficients, 95% Confidence Intervals, 95% CI) for step increases in exposure in the study, 1990–2010. Table D. Part A. Minimally adjusted hazard ratios for As_I_ indicator on the main mortality causes (HR, 95% Confidence Intervals, 95% CI) in the study, 1990–2010. Part B. Minimally adjusted hazard ratios for CAI indicator on the main mortality causes (HR, 95% Confidence Intervals, 95% CI) in the study, 1990–2010. Fig A. Box plots illustrating the distribution of As_I_ (μg/L) over the three exposure categories in the study subjects, 1990–2010. Boxes represent the interquartile range (25th–75th percentiles; median indicated by horizontal line), and whiskers extend to the 5th and 95th percentiles.
(DOC)

## Abbreviations

As: Arsenic
EU: European Union
As_I_: average arsenic concentration at individual level
CAI: cumulative arsenic dose indicator
LDI: Individual Daily Intake
HR: Hazard Ratio
COPD: Chronic Obstructive Pulmonary Disease
BMI: Body mass index
WHO: World Health Organization
EFSA: European Food Safety Authority
ICC: Intraclass correlation coefficient
EPA: US Environmental Protection Agency
