# Supplementary material for: Arsenic in Drinking Water and Mortality for Cancer and Chronic Diseases in Central Italy, 1990-2010
Source: PLoS One. 2015 Sep 18;10(9):e0138182. doi: 10.1371/journal.pone.0138182 (PMC4575137; doi:10.1371/journal.pone.0138182)
Supplement: S1 File — Boxes represent the interquartile range (25th–75th percentiles; median indicated by horizontal line), and whiskers extend to the 5th and 95th percentiles. (DOC) [file pone.0138182.s001.doc]

**Table A. Health status indicators included in the study, related evidence from epidemiological studies on populations exposed to low-moderate arsenic doses (<10**0 µg/L) in drinking water and possible underlying mechanisms.

| **SENSITIVE INDICATORS (ICD-9)** | **EPIDEMIOLOGICAL EVIDENCE** | | **EXPERIMENTAL EVIDENCE** | |
| --- | --- | --- | --- | --- |
|  | **RELEVANT REFERENCES** | **SUMMARY OF EFFECTS OF ARSENIC EXPOSURE** | **RELEVANT REFERENCES** | **POSSIBLE MECHANISMS LINKED TO ARSENIC EXPOSURE** |
| **Malignant cancers (140-208)** |  |  |  |  |
| Tracheas, bronchus and lung cancer (162) | SR [1-3] S [4] | - causal association at high doses but limited and uncertain evidence at low doses. - linear dose-response with cancer mortality in a cohort in USA. | S [5] | Not completely understood mechanisms; DNA damage in lung epithelium cells due to oxidative stress for the high lung partial pressure or due to free radical production induced by gas dymethilarsine. |
| Bladder cancer (188) | SR [2, 3, 6] | - causal association at high doses but limited evidence at low doses. | S [7, 8] | Epigenetic alterations as reduced hystone acetylation in bladder epithelium cells and tumor suppressor gene promoter hypermethylation. |
| Cancers of liver and bile ducts (155, 156) | SR [3] | - positive association but limited evidence at high and low doses. | R [9] | Not completely understood mechanisms; genomic instability in hepatic cells due to epigenetic alterations, i.e. global loss of DNA methylation. |
| Prostate cancer (185) | SR [3] S [10] | - positive association but limited evidence at high and low doses. - linear dose-response with cancer mortality in a cohort in USA. | R [11] | Enhanced prostate epithelial cells proliferation due to DNA hypomethylation and overexpression of K-ras that codifies for a intramembrane protein involved in signalling to androgens. |
| Kidney cancer (189) | SR [3] | - positive association but limited evidence at high and low doses. | S [12] | Renal cells proliferation due to inactivation of tumor suppressor gene p53 due to genetic polymorphism or overexpression of oncogene mdm2 levels with disruption of cell cycle. |
| **Diseases of the circulatory system (390-459)** |  |  |  |  |
| Ischemic heart disease (410-414) | SR [13]  S [14, 15] | - positive association at high doses but insufficient evidence at low doses and low quality studies. - linear dose-response with mortality and incidence of coronary heart disease, and stroke in USA and Spain. | R [18, 19] | Atherosclerotic damage in vascular endothelial cells due to reduced fibrinolytic activity, release of pro-inflammatory cytokines, smooth muscle cell proliferation, oxidative stress and endothelial injury.  Vascular endothelial dysfunction due to reduced nitric oxide, endothelial citotoxicity, increased oxidative stress and decreased endothelium-dependent vasorelaxation. |
| Myocardial infarction (410) | S [16, 17] |  |  |  |
| Coronary atherosclerosis (414) | S [17] |  |  |  |
| Cerebrovascular diseases (430-438) | SR [13]  S [14, 15] |  |  |  |
| Stroke (430, 431, 434, 436) | S [16] |  |  |  |
| Peripheral Arterial Disease (440-448) | SR [13] |  |  |  |
| **Diseases of the respiratory system (460-519)** |  |  |  |  |
| COPD (490-496) | S [20, 21] | - scarce evidence at high and low doses of association with respiratory symptoms and lung function impairment. | S [22, 23] | Unknown mechanisms. Decreased immune response to respiratory infections, i.e. immediate decrease in dendritic cells in the mediastinal lymphonodes. Damage on lung’s alveolar epithelium (i.e. Clara cells), and reduced secretion of the anti-inflammatory CC16 protein. |
| Diabetes mellitus (250) | SR [24, 25]  S [26, 27] | - limited-sufficient evidence of causal association at high doses but insufficient evidence at low doses except few studies with better measured outcomes and exposure. - positive association with diabetes in USA and Serbia. | R [28]  S [29, 30] | Inconclusive evidence on mechanisms. Oxidative stress-mediated damage in pancreatic cells and impairment of glucose-stimulated insulin secretion from pancreatic β-cells. Insulin resistance in cells of peripheral tissues (i.e. inhibition of intracellular signalling or glucose uptake, or by impairment of cell differentiation). |

R: narrative review; S: single study; SR: systematic review

**References**

1. Celik I, Gallicchio L, Boyd K, Lam TK, Matanoski G, et al. (2008) Arsenic in drinking water and lung cancer: a systematic review. Environ Res 108: 48-55. http://dx.doi.org/10.1016/j.envres.2008.04.001.
2. Begum M, Horowitz J, Hossain MI. (2012) Low-dose risk assessment for arsenic: a meta-analysis approach. Asia Pac J Public Health 1010539512466568.
3. IARC Working Group on the Evaluation of Carcinogenic Risks to Humans. (2012) Arsenic, metals, fibres, and dusts. IARC Monogr Eval Carcinog Risks Hum 100(Pt C): 11-465. Available: <http://monographs.iarc.fr/ENG/Monographs/vol100C/mono100C.pdf>. Accessed 26 June 2014.
4. García-Esquinas E, Pollán M, Umans JG, Francesconi KA, Goessler W, et al. (2013) Arsenic exposure and cancer mortality in a US-based prospective cohort: the strong heart study. Cancer Epidemiol Biomarkers Prev 22: 1944-1953. http://dx.doi.org/10.1158/1055-9965.EPI-13-0234-T.
5. Kitchin KT, Conolly R. (2010) Arsenic-induced carcinogenesiss oxidative stress as a possible mode of action and future research needs for more biologically based risk assessment. Chem Res Toxicol 23: 327-335. http://dx.doi.org/10.1021/tx900343d.
6. Mink PJ, Alexander DD, Barraj LM, Kelsh MA, Tsuji JS. (2008) Low-level arsenic exposure in drinking water and bladder cancer: a review and meta-analysis. Regul Toxicol Pharmacol 52: 299-310. http://dx.doi.org/10.1016/j.yrtph.2008.08.010.
7. Marsit CJ, Karagas MR, Danaee H, Liu M, Andrew A, et al. (2006) Carcinogen exposure and gene promoter hypermethylation in bladder cancer. Carcinogenesis 27: 112-116.
8. Jo WJ, Ren X, Chu F, Aleshin M, Wintz H, et al. (2009) Acetylated H4K16 by MYST1 protects UROtsa cells from arsenic toxicity and is decreased following chronic arsenic exposure. Toxicol Appl Pharmacol 241(3): 294-302. http://dx.doi.org/10.1016/j.taap.2009.08.027.
9. Liu J, Waalkes MP. (2008) Liver is a target of arsenic carcinogenesis. Toxicol Sci 105: 24-32. http://dx.doi.org/10.1093/toxsci/kfn120.
10. García-Esquinas E, Pollán M, Umans JG, Francesconi KA, Goessler W, et al. (2013) Arsenic exposure and cancer mortality in a US-based prospective cohort: the strong heart study. Cancer Epidemiol. Biomarkers Prev 22: 1944-1953. http://dx.doi.org/10.1158/1055-9965.EPI-13-0234-T.
11. Benbrahim-Tallaa L, Waalkes MP. (2008) Inorganic arsenic and human prostate cancer. Environ Health Perspect 116: 158-164. <http://dx.doi.org/10.1289/ehp.10423>
12. Huang CY, Su CT, Chu JS, Huang SP, Pu YS, et al. (2011) The polymorphisms of P53 codon 72 and MDM2 SNP309 and renal cell carcinoma risk in a low arsenic exposure area. Toxicol Appl Pharmacol 257: 349-355. http://dx.doi.org/10.1016/j.taap.2011.09.018.
13. Moon K, Guallar E, Navas-Acien A. 2012. Arsenic exposure and cardiovascular disease: an updated systematic review. Curr Atheroscler Rep 14: 542-555. http://dx.doi.org/10.1007/s11883-012-0280-x.
14. Medrano MA, Boix R, Pastor-Barriuso R, Palau M, Damián J, et al. (2010) Arsenic in public water supplies and cardiovascular mortality in Spain. Environ Res 110: 448-454. http://dx.doi.org/10.1016/j.envres.2009.10.002.
15. Moon KA, Guallar E, Umans JG, Devereux RB, Best LG, et al. (2013) Association between exposure to low to moderate arsenic levels and incident cardiovascular disease. A prospective cohort study. Ann Intern Med 159: 649-659.
16. Zierold KM, Knobeloch L, Anderson H. (2004) Prevalence of chronic diseases in adults exposed to arsenic-contaminated drinking water. Am J Public Health 94: 1936-1937.
17. Meliker JR, Wahl RL, Cameron LL, Nriagu JO. (2007) Arsenic in drinking water and cerebrovascular disease, diabetes mellitus, and kidney disease in Michigan: a standardized mortality ratio analysis. Environ Health 6: 4.
18. Balakumar P, Kaur J. (2009) Arsenic exposure and cardiovascular disorders: an overview. Cardiovasc Toxicol 9: 169-176. <http://dx.doi.org/10.1007/s12012-009-9050-6>.
19. Wu F, Molinaro P, Chen Y. Arsenic Exposure and Subclinical Endpoints of Cardiovascular Diseases. Curr Environ Health Rep. 2014;1: 148-162.
20. Parvez F, Chen Y, Brandt-Rauf PW, Slavkovich V, Islam T, et al. (2010) A prospective study of respiratory symptoms associated with chronic arsenic exposure in Bangladesh: findings from the Health Effects of Arsenic Longitudinal Study (HEALS). Thorax 65: 528-533. http://dx.doi.org/10.1136/thx.2009.119347.
21. Parvez F, Chen Y, Yunus M, Olopade C, Segers S, et al. (2013) Arsenic exposure and impaired lung function. Findings from a large population-based prospective cohort study. Am J Respir Crit Care Med 188: 813-819. http://dx.doi.org/10.1164/rccm.201212-2282OC.
22. Kozul CD, Ely KH, Enelow RI, Hamilton JW. (2009) Low-dose arsenic compromises the immune response to influenza A infection in vivo. Environ Health Perspect 117: 1441-1447. http://dx.doi.org/10.1289/ehp.0900911.
23. Parvez F, Chen Y, Brandt-Rauf PW, Bernard A, Dumont X, et al. (2008) Nonmalignant respiratory effects of chronic arsenic exposure from drinking water among never-smokers in Bangladesh. Environ Health Perspect 116: 190-195. http://dx.doi.org/10.1289/ehp.9507.
24. Maull EA, Ahsan H, Edwards J, Longnecker MP, Navas-Acien A, et al. (2012) Evaluation of the association between arsenic and diabetes: A National Toxicology Program Workshop Review. Environ Health Perspect 120: 1658-1670. http://dx.doi.org/10.1289/ehp.1104579.
25. Wang W, Xie Z, Lin Y, Zhang D. (2014) Association of inorganic arsenic exposure with type 2 diabetes mellitus: a meta-analysis. J Epidemiol Community Health 68: 176-184. http://dx.doi.org/10.1136/jech-2013-203114.
26. James KA, Marshall JA, Hokanson JE, Meliker JR, Zerbe GO, et al. (2013) A case-cohort study examining lifetime exposure to inorganic arsenic in drinking water and diabetes mellitus. Environ Res 123: 33-38. http://dx.doi.org/10.1016/j.envres.2013.02.005.
27. Jovanovic D, Rasic-Milutinovic Z, Paunovic K, Jakovljevic B, Plavsic S, et al. (2013) Low levels of arsenic in drinking water and type 2 diabetes in Middle Banat region, Serbia. Int J Hyg Environ Health 216: 50-55. http://dx.doi.org/10.1016/j.ijheh.2012.01.001.
28. Huang CF, Chen YW, Yang CY, Tsai KS, Yang RS, et al. (2011) Arsenic and diabetes: Current perspectives. Kaohsiung J Med Sci 27: 402-410. http://dx.doi.org/10.1016/j.kjms.2011.05.008.
29. Walton FS, Harmon AW, Paul DS, Drobna Z, Patel YM, et al. (2004) Inhibition of insulin-dependent glucose uptake by tri­valent arsenicals: possible mechanism of arsenic-induced diabetes. Toxicol Appl Pharmacol 198: 424–433.
30. Yen YP, Tsai KS, Chen YW, Huang CF, Yang RS, et al. (2010) Arsenic inhibits myogenic differentiation and muscle regen­eration. Environ Health Perspect 118: 949-956. http://dx.doi.org/10.1289/ehp.0901525.

**Table B. Associations of individual daily intake (LDI) and mortality from specific causes (HR, 95% Confidence Intervals, 95% CI) in the study, 1990-2010.**

|  | **LDI (µg per Kg b.w./day)** | | | | | | |  |
| --- | --- | --- | --- | --- | --- | --- | --- | --- |
| **Causes of death (ICD-9)** | **(≤ 0.113)** |  |  | **(0.113 -0.429)** |  |  | **(> 0.429)** | ***p*-value for trend** |
|  | **Deaths** |  | **Deaths** | **HR^a,b^ (95% CI)** |  | **Deaths** | **HR^a,b^ (95% CI)** |
| **Males (N=68758)** |  |  |  |  |  |  |  |  |
| Natural causes (001-799) | 4397 |  | 5408 | 1.00 (0.93, 1.08) |  | 1009 | **1.45 (1.30, 1.63)** | **<0.001** |
| Malignant cancers (140-208) | 1346 |  | 1661 | 0.98 (0.87, 1.12) |  | 353 | **1.62 (1.34, 1.97)** | **<0.001** |
| Liver and bile ducts (155, 156) | 83 |  | 101 | 0.97 (0.66, 1.43) |  | 23 | **2.05 (1.10, 3.83)** | 0.112 |
| Tracheas, bronchus and lung (162) | 378 |  | 529 | 1.02 (0.82, 1.26) |  | 104 | **1.72 (1.22, 2.41)** | **0.014** |
| Prostate (185) | 102 |  | 124 | 1.15 (0.87, 1.51) |  | 21 | 1.05 (0.65, 1.69) | 0.484 |
| Bladder (188) | 58 |  | 70 | 1.20 (0.77, 1.87) |  | 29 | **3.35 (1.74, 6.47)** | **0.002** |
| Kidney (189) | 31 |  | 31 | 0.96 (0.53, 1.73) |  | 6 | 1.11 (0.39, 3.12) | 0.956 |
| Circulatory system diseases (390-459) | 1650 |  | 2000 | 1.04 (0.93, 1.16) |  | 387 | **1.55 (1.29, 1.86)** | **<0.001** |
| Ischemic heart disease (410-414) | 488 |  | 645 | 1.08 (0.89, 1.31) |  | 124 | **1.68 (1.22, 2.30)** | **0.007** |
| Myocardial infarction (410) | 251 |  | 323 | 1.17 (0.91, 1.49) |  | 69 | **1.85 (1.23, 2.77)** | **0.008** |
| Coronary atherosclerosis (414) | 225 |  | 299 | 1.01 (0.77, 1.32) |  | 54 | 1.53 (0.97, 2.41) | 0.178 |
| Cerebrovascular diseases (430-438) | 454 |  | 538 | 1.15 (0.94, 1.41) |  | 86 | 1.28 (0.90, 1.84) | 0.110 |
| Stroke (430, 431, 434, 436) | 361 |  | 445 | **1.29 (1.03, 1.61)** |  | 71 | **1.52 (1.02, 2.26)** | **0.013** |
| Peripheral Arterial (440-448) | 133 |  | 139 | 0.88 (0.62, 1.26) |  | 24 | 1.46 (0.74, 2.90) | 0.827 |
| Respiratory system diseases (460-519) | 246 |  | 440 | 1.00 (0.76, 1.32) |  | 60 | 1.07 (0.69, 1.65) | 0.818 |
| COPD (490-496) | 110 |  | 177 | 1.09 (0.76, 1.55) |  | 32 | 1.25 (0.69, 2.25) | 0.457 |
| Diabetes mellitus (250) | 86 |  | 110 | 0.83 (0.54, 1.28) |  | 27 | 0.98 (0.50, 1.91) | 0.822 |
|  |  |  |  |  |  |  |  |  |
| **Females (N=70042)** |  |  |  |  |  |  |  |  |
| Natural causes (001-799) | 2512 |  | 5899 | **1.43 (1.32, 1.55)** |  | 1551 | **1.76 (1.58, 1.97)** | **<0.001** |
| Malignant cancers (140-208) | 526 |  | 1433 | **1.54 (1.32, 1.79)** |  | 367 | **1.94 (1.57, 2.40)** | **<0.001** |
| Liver and bile ducts (155, 156) | 31 |  | 100 | **2.03 (1.02, 4.05)** |  | 25 | **2.88 (1.16, 7.12)** | **0.024** |
| Tracheas, bronchus and lung (162) | 48 |  | 142 | **1.64 (1.12, 2.40)** |  | 42 | **2.62 (1.57, 4.40)** | **<0.001** |
| Bladder (188) | 13 |  | 20 | 0.95 (0.43, 2.14) |  | 5 | 0.93 (0.26, 3.31) | 0.898 |
| Kidney (189) | 7 |  | 17 | 1.48 (0.54, 4.03) |  | 7 | 3.64 (0.98, 13.55) | 0.069 |
| Circulatory system diseases (390-459) | 1029 |  | 2619 | **1.51 (1.34, 1.70)** |  | 696 | **1.92 (1.63, 2.27)** | **<0.001** |
| Ischemic heart disease (410-414) | 213 |  | 639 | **1.70 (1.33, 2.17)** |  | 162 | **2.07 (1.48, 2.86)** | **<0.001** |
| Myocardial infarction (410) | 83 |  | 250 | **1.69 (1.20, 2.37)** |  | 64 | **2.30 (1.43, 3.72)** | **<0.001** |
| Coronary atherosclerosis (414) | 128 |  | 377 | **1.66 (1.23, 2.22)** |  | 98 | **1.93 (1.29, 2.89)** | **0.001** |
| Cerebrovascular diseases (430-438) | 312 |  | 753 | **1.43 (1.19, 1.72)** |  | 198 | **1.95 (1.48, 2.56)** | **<0.001** |
| Stroke (430, 431, 434, 436) | 263 |  | 605 | **1.37 (1.13, 1.68)** |  | 152 | **1.70 (1.27, 2.28)** | **<0.001** |
| Peripheral Arterial (440-448) | 64 |  | 181 | 1.41 (0.93, 2.13) |  | 39 | 1.36 (0.73, 2.53) | 0.237 |
| Respiratory system diseases (460-519) | 92 |  | 258 | **1.50 (1.08, 2.10)** |  | 69 | **1.71 (1.08, 2.72)** | **0.018** |
| COPD (490-496) | 33 |  | 99 | 1.53 (0.96, 2.44) |  | 25 | 1.73 (0.90, 3.33) | 0.084 |
| Diabetes mellitus (250) | 73 |  | 227 | **1.92 (1.33, 2.78)** |  | 79 | **2.38 (1.47, 3.84)** | **<0.001** |

a HR: Hazard Ratios and 95%CI calculated respect to LDI≤25° pct (0.113 µg per Kg b.w./day) as reference group; significant HR and p-values for trend highlighted in bold

b Models adjusted for age, calendar period, socioeconomic level, occupation in the ceramic industry and radon exposure

**Table C. Effects of average arsenic concentrations at individual level at the first year of residence (As_I_**) and mortality for specific causes (beta coefficients, 95% Confidence Intervals, 95% CI) for step increases in exposure in the study, 1990-2010.

| Step increases in As_I_(µg/L) | Mortality for lung cancer (ICD-9: 162) | | |  | Mortality for cardiovascular disease (ICD-9: 390-459) | | |
| --- | --- | --- | --- | --- | --- | --- | --- |
|  | Betacoefficient | 95% CI | |  | Betacoefficient | 95% CI | |
| 0 vs 1 | -0.281 | -0.698 | 0.137 |  | -0.323 | -0.484 | -0.161 |
| 1 vs 2 | -0.105 | -0.355 | 0.145 |  | -0.141 | -0.237 | -0.044 |
| 2 vs 3 | 0.027 | -0.097 | 0.152 |  | -0.002 | -0.050 | 0.046 |
| 3 vs 4 | 0.116 | 0.058 | 0.175 |  | 0.092 | 0.070 | 0.113 |
| 4 vs 5 | 0.162 | 0.094 | 0.229 |  | 0.143 | 0.118 | 0.168 |
| 5 vs 6 | 0.164 | 0.086 | 0.241 |  | 0.149 | 0.120 | 0.178 |
| 6 vs 7 | 0.131 | 0.068 | 0.193 |  | 0.121 | 0.097 | 0.144 |
| 7 vs 8 | 0.095 | 0.049 | 0.140 |  | 0.088 | 0.071 | 0.106 |
| 8 vs 9 | 0.064 | 0.032 | 0.096 |  | 0.061 | 0.049 | 0.073 |
| 9 vs 10 | 0.039 | 0.016 | 0.062 |  | 0.039 | 0.030 | 0.048 |
| 10 vs 15 | 0.011 | -0.081 | 0.102 |  | 0.024 | -0.012 | 0.061 |
| 15 vs 20 | 0.005 | -0.056 | 0.066 |  | 0.011 | -0.012 | 0.035 |
| 20 vs 25 | 0.050 | 0.015 | 0.084 |  | 0.044 | 0.030 | 0.058 |
| 25 vs 30 | 0.087 | 0.043 | 0.131 |  | 0.070 | 0.052 | 0.089 |
| 30 vs 35 | 0.117 | 0.054 | 0.179 |  | 0.090 | 0.065 | 0.116 |
| 35 vs 40 | 0.139 | 0.064 | 0.213 |  | 0.104 | 0.073 | 0.134 |
| 40 vs 45 | 0.153 | 0.075 | 0.232 |  | 0.111 | 0.079 | 0.143 |
| 45 vs 50 | 0.160 | 0.088 | 0.233 |  | 0.112 | 0.082 | 0.141 |
| 50 vs 55 | 0.160 | 0.101 | 0.218 |  | 0.106 | 0.083 | 0.130 |
| 55 vs 60 | 0.152 | 0.113 | 0.190 |  | 0.094 | 0.079 | 0.110 |
| 60 vs 65 | 0.136 | 0.104 | 0.168 |  | 0.076 | 0.063 | 0.089 |
| 65 vs 70 | 0.113 | 0.048 | 0.178 |  | 0.051 | 0.024 | 0.078 |
| 70 vs 75 | 0.082 | -0.036 | 0.200 |  | 0.020 | -0.028 | 0.069 |
| 75 vs 80 | 0.043 | -0.139 | 0.226 |  | -0.017 | -0.092 | 0.058 |
| 80 vs 85 | -0.002 | -0.261 | 0.256 |  | -0.061 | -0.167 | 0.045 |

**Table D**

**Part D1 - Minimally adjusted hazard ratios for As_I_** indicator on the main mortality causes (HR, 95% Confidence Intervals, 95% CI) in the study, 1990-2010.

| **Causes of death (ICD-9)** | **AsI (µg/L)** | | | | | | |
| --- | --- | --- | --- | --- | --- | --- | --- |
|  | **≤10^a^** |  | **10-20^a^** | |  | **>20^a^** | |
|  | **Deaths** |  | **Deaths** | **HR^b,c^ (95% CI)** |  | **Deaths** | **HR^b,c^ (95% CI)** |
| **Males (N=68758)** |  |  |  |  |  |  |  |
| Tracheas, bronchus and lung cancer (162) | 283 |  | 259 | 0.99 (0.81, 1.21) |  | 469 | **1.68 (1.40, 2.05)** |
| Diseases of the circulatory system (390-459) | 1317 |  | 1061 | 0.93 (0.83, 1.03) |  | 1659 | **1.46 (1.28, 1.66)** |
| Diseases of the respiratory system (460-519) | 190 |  | 163 | 1.04 (0.81, 1.35) |  | 393 | **1.86 (1.43, 2.41)** |
| Diabetes mellitus (250) | 67 |  | 46 | 0.79 (0.52, 1.21) |  | 110 | **1.57 (1.06, 2.30)** |
|  |  |  |  |  |  |  |  |
| **Females (N=70042)** |  |  |  |  |  |  |  |
| Tracheas, bronchus and lung cancer (162) | 63 |  | 69 | 1.22 (0.85, 1.74) |  | 100 | **1.53 (1.09, 2.15)** |
| Diseases of the circulatory system (390-459) | 1435 |  | 1054 | 0.88 (0.80, 0.98) |  | 1855 | **1.34 (1.19, 1.50)** |
| Diseases of the respiratory system (460-519) | 127 |  | 107 | 1.06 (0.79, 1.43) |  | 185 | **1.43 (1.07, 1.90)** |
| Diabetes mellitus (250) | 95 |  | 84 | 1.14 (0.82, 1.59) |  | 200 | **2.08 (1.53, 2.54)** |

^a^ Person time of exposure of individual study subjects in the three As_I_ exposure categories: Males: As_I_ ≤10 µg/L: n=379,421; As_I_=10-20 µg/L: n=350,192; As_I_>20 µg/L: n=442,993; Females: As_I_ ≤10 µg/L: n=392,439; As_I_=10-20 µg/L: n=363,084; As_I_>20 µg/L: n=461,136

^b^ HR: Hazard Ratios and 95%CI calculated respect to As≤10 μg/L as reference group; significant HR and p-values for trend highlighted in bold

^c^ Models adjusted for age and calendar period

**Part D2 -** Minimally adjusted hazard ratios for CAI indicator on the main mortality causes (HR, 95% Confidence Intervals, 95% CI) in the study, 1990-2010.

| **Causes of death (ICD-9)** | **Cumulative arsenic index (µg)** | | | | | | |
| --- | --- | --- | --- | --- | --- | --- | --- |
|  | **25° pct** |  | **25° -75° pct** | |  | **75° pct** | |
|  | **(≤ 204.9 µg)^a^** |  | **(204.9 -804.0 µg)^a^** | |  | **(> 804.0 µg)^a^** | |
|  | **Deaths** |  | **Deaths** | **HR^a,b^ (95% CI)** |  | **Deaths** | **HR^a,b^ (95% CI)** |
| **Males (N=68758)** |  |  |  |  |  |  |  |
| Tracheas, bronchus and lung cancer (162) | 53 |  | 419 | **1.91 (1.43, 2.55)** |  | 539 | **2.39 (1.78, 3.21)** |
| Diseases of the circulatory system (390-459) | 242 |  | 1720 | **1.63 (1.42, 1.88)** |  | 2075 | **1.80 (1.55, 2.09)** |
| Diseases of the respiratory system (460-519) | 35 |  | 260 | **1.57 (1.09, 2.27)** |  | 451 | **1.78 (1.20, 2.63)** |
| Diabetes mellitus (250) | 15 |  | 87 | 1.32 (0.74, 2.35) |  | 121 | 1.51 (0.83, 2.75) |
|  |  |  |  |  |  |  |  |
| **Females (N=70042)** |  |  |  |  |  |  |  |
| Tracheas, bronchus and lung cancer (162) | 15 |  | 98 | 1.69 (0.98, 2.91) |  | 119 | **1.97 (1.14, 3.39)** |
| Diseases of the circulatory system (390-459) | 241 |  | 1732 | **1.71 (1.48, 1.98)** |  | 2371 | **1.61 (1.38, 1.88)** |
| Diseases of the respiratory system (460-519) | 31 |  | 150 | 1.10 (0.72, 1.66) |  | 238 | 1.17 (0.76, 1.80) |
| Diabetes mellitus (250) | 17 |  | 120 | 1.66 (0.98, 2.82) |  | 242 | **2.66 (1.55, 4.56)** |

^a^ Person time of exposure of individual study subjects in the three CAI exposure categories: Males: CAI≤ 204.9 µg: n=296,778; CAI=204.9-804.0 µg: n=595,344; CAI>804.0 µg: n=282,942; Females: CAI≤ 204.9 µg: n=294,332; CAI=204.9-804.0 µg: n=607,538; CAI>804.0 µg: n=317,308

^b^ HR: Hazard Ratios and 95%CI calculated respect to CAI ≤25° pct (204.9 μg) as reference group; significant HR and p-values for trend highlighted in bold

^c^ Models adjusted for age and calendar period

**Fig A.** Box plots illustrating the distribution of As_I_ (μg/L) over the three exposure categories in the study subjects, 1990-2010. Boxes represent the interquartile range (25th–75th percentiles; median indicated by horizontal line), and whiskers extend to the 5th and 95th percentiles.
